# Proton transport modeling in a realistic biological environment by using *TILDA-V*

**DOI:** 10.1038/s41598-019-50270-5

**Published:** 2019-10-01

**Authors:** Mario E. Alcocer-Ávila, Michele A. Quinto, Juan M. Monti, Roberto D. Rivarola, Christophe Champion

**Affiliations:** 1 CELIA, Centre Lasers Intenses et Applications, Université de Bordeaux – CNRS – CEA, F-33400 Talence, France; 2 Instituto de Física Rosario, CONICET – Universidad Nacional de Rosario, S2000 EKF Rosario, Argentina

**Keywords:** Biological physics, Quantum physics

## Abstract

Whether it is in radiobiology to identify DNA lesions or in medicine to adapt the radiotherapeutic protocols, a detailed understanding of the radiation-induced interactions in living matter is required. Monte Carlo track-structure codes have been successfully developed to describe these interactions and predict the radiation-induced energy deposits at the nanoscale level in the medium of interest. In this work, the quantum-mechanically based Monte Carlo track-structure code *TILDA-V* has been used to compute the slowing-down of protons in water and DNA. Stopping power and range are then reported and compared with existing data. Then, a first application of *TILDA-V* to cellular irradiations is also reported in order to highlight the absolute necessity of taking into account a realistic description of the cellular environment in microdosimetry.

## Introduction

Radiation physics remains nowadays an active field of research. On the one hand, ionizing radiations are widely used in the clinical setting for diagnostic and therapeutic purposes, being one of the most effective tools to cure cancer^1^. Moreover, its use will likely continue to grow in importance since new promising medical techniques to fight cancer are currently under study, some of which are based in the improved therapeutic outcome of combining radiotherapy with immunotherapy^2,3^. On the other hand, a precise knowledge of the mechanisms through which ionizing radiations induce damage on the DNA molecule is still of great importance for understanding the effects of low radiation doses on human health, either for radiation protection measures or for enhancing the effectiveness of cancer treatments. In this context, Monte Carlo track-structure (MCTS) codes are powerful tools able to tackle various problems in radiotherapy and radiobiology, including the estimation of microdosimetric parameters with a high level of accuracy^4^. Several MCTS codes have been developed over the last three decades to simulate the full slowing-down of particles in matter. These codes are frequently based on a combination of semi-empirical and theoretical interaction models where water is commonly used as the target, since it has long been considered as an appropriate substitute for biological soft tissue. The most sophisticated platforms are able to simulate the physical, physicochemical and chemical stages of water radiolysis to predict the direct and indirect effects of ionizing radiation on the DNA molecule. However, this is achieved by superimposing a specific DNA geometry on the radiation tracks computed in water. Some well-known examples of MCTS codes in continuous development are GEANT4-DNA^5,6^, KURBUC^7,8^ and PARTRAC^9,10^. The latter is a state-of-the-art MCTS code that also considers a DNA double strand break repair model via the non-homologous end-joining pathway. The main differences between the aforementioned tools and *TILDA-V*, the MCTS code used in this paper, is that *TILDA-V* largely relies on differential and total cross sections computed *ab initio* within a quantum mechanical treatment of interactions and applying a molecular description of both water and DNA, as explained hereafter. For a comprehensive review on the subject of radiation track-structure and the existing MCTS codes the reader is referred to the works of Nikjoo *et al*.^4,11^.

Furthermore, it is worth mentioning that track-structure simulations are not the only computational approach implemented in recent years to investigate the DNA damage induced by ions. For instance, the Monte Carlo Damage Simulation (MCDS) code^12,13^ is a fast tool that estimates the number of simple and complex DNA lesions produced by electrons and light ions. The MCDS algorithm randomly distributes an expected number of lesions in a DNA segment and then groups the lesions into damage clusters. MCDS requires four adjustable input parameters, which are based on interpolated damage yields derived from track-structure simulations. Three of these parameters, namely the number of individual strand breaks per unit dose per cell, the base damage to strand break ratio and the minimum length of undamaged DNA between neighboring elementary damages, are considered as independent of radiation quality. The fourth parameter is the DNA segment length and depends on particle type and kinetic energy^12^. A computer code using the output of the MCDS algorithm to simulate the excision repair of single and clustered DNA damages, other than double strand breaks, has been reported as well by Semenenko *et al*.^14^.

The purpose of this work is to highlight the fact that important differences may arise when estimating several physical quantities related to the transport of protons in living matter, depending on how the biological medium is modeled and whether or not the description goes beyond the common assumption of taking water as a surrogate for tissue. To do so, we report some macroscopic quantities concerning the transport of protons in water and DNA, as provided by our MCTS code, *TILDA-V*. Firstly, a comparison of the stopping power and range values computed for protons in both media is presented. Then, an example of the application of *TILDA-V* to radiation microdosimetry in individual cells is described in detail.

## Materials and Methods

Monte Carlo (MC) simulations were carried out using *TILDA-V*, a *homemade* Monte Carlo track-structure code designed to describe the transport of light ions in biological matter. In its latest version, *TILDA-V* is able to follow protons with kinetic energies in the range from 10 keV to 100 MeV, as well as the secondary electrons produced along the primary particle’s trajectory down to 7.4 eV.

A remarkable feature of *TILDA-V* is that it implements a set of *ab initio* multiple differential and total cross sections (TCS), calculated within the quantum mechanical framework, for describing the main inelastic processes during the slowing-down of protons in water and DNA. These cross sections are directly related to the probability of a given physical process to occur and reflect the stochastic nature of radiation transport. Moreover, within *TILDA-V*, the cross sections constitute the basic input for performing the simulations and all macroscopic quantities are computed from proton transport simulations. Currently the interactions taken into account in the code are: ionization, excitation, electron capture and elastic scattering by protons; ionization, excitation, electron loss and elastic scattering by neutral hydrogen. For the present calculations the models listed in Table 1 were used. A brief discussion of the models shown in Table 1 is presented in  the Theory section. A more detailed description of the *TILDA-V* code and the theoretical approach used for describing each collisional process as well as the targets has been given elsewhere^15^.

**Table 1:** Set of models used in *TILDA-V* for the present calculations.

	Process	Model
Proton	Ionization	*Prior* CDW-EIS^83^
	Capture	*Prior* CDW-EIS^47^
	Excitation	Miller and Green^15,21,49^
	Elastic scattering	Classical description^84^
Hydrogen	Ionization	*Prior* CDW-EIS^43^
	Excitation	Miller and Green^15,49,53^
	Electron loss	Miller and Green^15,49^
	Elastic scattering	Classical description^54^
Electron	Ionization	DWBA^85^
	Excitation	Olivero *et al*.^86^
	Elastic scattering	Partial wave formalism^87^

CDW-EIS: Continuum distorted wave-eikonal initial state approximation; DWBA: Distorted-wave Born approximation.

It should be noted that *TILDA-V* only simulates the physical stage of energy deposition in biological targets, necessary to account for the direct effects of ionizing radiation on matter. Therefore, the creation and subsequent interaction of free radical species with the DNA molecule, i.e. the indirect effects of radiation, is not presently included in the code. We are aware that the chemical stage should not be disregarded in a detailed analysis of DNA damages, but this is beyond the scope of this work, which is limited to a general dosimetric study. Besides, nuclear non-elastic scattering is currently not included in the simulations.

The tracking of the secondary electrons emitted in the various ionizing processes is performed with a MCTS code called *EPOTRAN*, which is already integrated into *TILDA-V*. For the details about this code and the interactions of the secondary particles we refer the reader to the previous works by Champion *et al*.^16,17^.

Let us emphasize that, in contrast to some other Monte Carlo codes, in *TILDA-V* the stopping power (SP) is an outcome computed independently from other simulations and therefore it is not part of the input data. The SP is obtained by simulating one million projectiles (both protons and neutral hydrogen) in stationary mode for each incident energy. In this mode, each primary particle is followed until it experiences an interaction with the medium. A sum over the energy loss and the distance traveled by each particle is carried out and the stopping power – expressed in keV/*μ*m – is then defined as

1 $$SP=\frac{{E}_{{\rm{tot}}}}{L},$$

where *E*_tot_ is the total energy lost by the stationary projectiles and *L* is the track length. Taking into account the relative contributions of the proton and neutral hydrogen, the total stopping power can be written as

2 $$SP={f}_{{{\rm{H}}}^{+}}{(SP)}_{{{\rm{H}}}^{+}}+{f}_{{{\rm{H}}}^{0}}{(SP)}_{{{\rm{H}}}^{0}},$$

where ${(SP)}_{{{\rm{H}}}^{+}}$ and ${(SP)}_{{{\rm{H}}}^{0}}$ refer to the electronic stopping power for the charged and neutral beams, respectively. The factors${f}_{{{\rm{H}}}^{+}}$ and ${f}_{{{\rm{H}}}^{0}}$ are the corresponding equilibrium charge fractions which can be written as^18^

3 $${f}_{{{\rm{H}}}^{+}}=\frac{{\sigma }_{{\rm{L}}}}{{\sigma }_{{\rm{L}}}+{\sigma }_{{\rm{C}}}}$$

and

4 $${f}_{{{\rm{H}}}^{0}}=\frac{{\sigma }_{{\rm{C}}}}{{\sigma }_{{\rm{L}}}+{\sigma }_{{\rm{C}}}},$$

where *σ*_L_ and *σ*_C_ denote the TCS for electron loss and electron capture, respectively.

The total inelastic mean free path (IMFP) can be written as

5 $$IMFP=\frac{1}{n{\sigma }_{T}},$$

where *n* denotes the number of target molecules per volume unit defined as

6 $$n=\frac{{N}_{A}\rho }{{A}_{{\rm{mol}}}},$$

where *N*_*A*_ is the Avogadro’s number, *ρ* and *A*_mol_ are the density (in g cm^−3^) and the molar mass of the crossed medium, respectively. *σ*_*T*_ denotes the TCS including all inelastic interactions considered to model the transport of H^+^ and H^0^ taking into account the relative contribution of each charge state, and is given by

7 $${\sigma }_{T}={f}_{{{\rm{H}}}^{+}}{({\sigma }_{T})}_{{{\rm{H}}}^{+}}+{f}_{{{\rm{H}}}^{0}}{({\sigma }_{T})}_{{{\rm{H}}}^{0}},$$

where ${({\sigma }_{T})}_{{{\rm{H}}}^{+}}$ and ${({\sigma }_{T})}_{{{\rm{H}}}^{0}}$ denote the TCS for protons and neutral hydrogen atoms, respectively, and ${f}_{{{\rm{H}}}^{+}}$, ${f}_{{{\rm{H}}}^{0}}$ are the equilibrium charge fractions defined in Eqs 3 and 4, respectively.

To compute the proton range, the simulations are carried out in slowing-down mode, i.e. each primary particle is followed until its energy falls below the fixed cutoff of 10 keV. The range is then obtained by adding all the distances traveled by the primary particles and dividing by the number of simulated projectiles.

**Figure 1:**
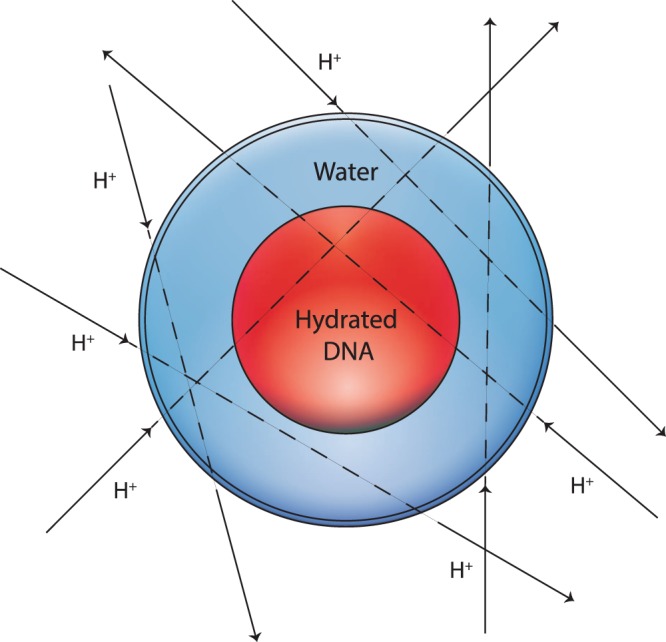
Irradiation of a single cell impacted by protons arriving at random directions. The innermost sphere colored in red represents the cell nucleus, here assumed to contain only hydrated DNA. The middle and outermost spherical shells colored in blue correspond to the cytoplasm and the cell membrane, respectively, and they are assumed to contain only water.

Finally, a radiation microdosimetry case-study was performed by considering a single cell with a simple geometry: three concentric spheres representing the basic regions of a cell, namely, the nucleus, the cytoplasm and the cell membrane (see Fig. 1). In our simulations the cell radius, nuclear radius and membrane thickness were fixed to 7 *μ*m, 4 *μ*m and 10 nm, respectively^19,20^. With respect to the composition of the regions, the cytoplasm and the cell membrane were modeled by water with a density of 1 g cm^−3^. To study the variation in the radiation dose deposited inside the cell nucleus depending on its composition, three different simulations were carried out in slowing-down mode for each incident proton energy, assuming the nucleus contained: (1) water, (2) water with the density rescaled to 1.29 g cm^−3^ and (3) hydrated DNA. The radiation source consisted of protons impinging on the cell from random directions, as shown in Fig. 1. The energy deposited by the primary and secondary particles was scored in each sphere and the dose *D* to the nucleus was then calculated following the well-known definition

8 $$D=\frac{\Delta E}{\Delta m}=\frac{\Delta E}{\rho \Delta V},$$

where Δ*E* is the energy deposited in the mass Δ*m*, *ρ* is the density of the medium and Δ*V* is the volume in which the energy is deposited, i.e., here the volume of the cell nucleus.

## Theory

In this section we will discuss the way in which biological media are modeled within *TILDA-V* and we will summarize the theoretical and semi-empirical models used to describe the different interactions of protons and neutral hydrogen atoms impinging on water and DNA molecules. As mentioned before, the tracking of the secondary electrons is taken into account by *EPOTRAN*, a MCTS code coupled to *TILDA-V*, which has been described elsewhere^17^. Therefore we will restrict the discussion to the interactions induced by the primary particles.

### The target description

Regarding the biomolecular targets studied, *TILDA-V* is based on a cross section database, which refers to isolated molecules in vapor state. In this context, the present work clearly differs from existing studies on condensed matter (water or DNA), where the energy-loss function of realistic biological components, which is extracted from experimental data and interpolated for several energies to cover the paucity of measurements, is used to compute the inelastic cross sections, i.e. ionization and excitation (see for example the works by Dingfelder *et al*.^21^, Abril and co-workers^22,23^ and Emfietzoglou *et al*.^24^). However, whether it is for water or DNA, the available data – cross sections as well as macroscopic outcomes like ranges and stopping power – are exclusively measured in vapor phase and comparisons with the liquid homologous have been only rarely reported.

#### Water

In the quantum mechanical framework, the atoms or molecules are described like ground state wave functions. In this context, the self-consistent field (SCF) theory attempts to evaluate atomic or molecular orbitals using the full many-electron Hamiltonian and a single determinantal wave function^25^. The orbital energies are optimized using the variational principle that leads to a set of differential equations which are normally out of hand, in particular for the molecular case where most applications of the theory have used linear combinations of atomic orbitals (LCAO). Commonly in ion-molecule collisions, the initial state of the water molecule ground state wave function has been described as molecular orbital (MO-LCAO-SCF) or complete neglect of differential overlap (CNDO). In the first representation, also known as Moccia’s description, the molecular orbitals are expressed in terms of Slater-like functions all centered at a common origin, and in the optimization calculations the geometry of the water molecule is included^26^. Whereas the CNDO approach, proposed by Pople *et al*.^25^, is an approximation of the self-consistent field molecular orbital method (SFC-MO), in which the differential overlap between atomic orbitals on the same atom are neglected. It is worth mentioning that Moccia’s molecular description is better than the CNDO one, but the latter requires less computational effort. Nevertheless, it has been shown that the initial description of molecular orbitals does not influence significantly the cross sections calculation performed within the CDW-EIS approximation. This study was reported by Tachino and co-workers, who investigated the influence of the ground state representation of water in the case of ionization^27^. Consequently, in this work we will adopt the CNDO approach in the description of water vapor, for which the corresponding atomic effective electron populations and the molecular binding energies are reported in Table 2 ^28^. Finally, we can report the cross section for a given molecular orbital *k* as

9 $${\sigma }_{k}=\mathop{\sum }\limits_{j=1}^{{N}_{k}}\,{c}_{k,j}\,{\sigma }_{j}$$

where *N*_*k*_ is the number of atoms that describe each molecular orbital. *σ*_*j*_ is the atomic orbital cross section involved in its LCAO description and *c*_*k*,*j*_ is the corresponding atomic effective occupation electron number. The different ground state atomic orbitals are described using Roothaan-Hartree-Fock atomic wave functions^29^.

**Table 2:** Population and binding energies of the H_2_O molecular orbitals.

Molecular orbital	Population	Binding Energy *ε*_*i*_ (eV)
1a1	2.0 O_1*s*_	−539.7
2a1	1.48 O_2*s*_ + 0.52 H_1*s*_	−32.2
1b2	1.18 O_2*p*_ + 0.82 H_1*s*_	−18.4
3a1	0.22 O_2*s*_ + 1.44 O_2*p*_ + 0.34 H_1*s*_	−14.7
1b1	2.0 O_2*p*_	−12.6

#### DNA

In our previous work^30^, we have proposed an *ab initio* approach to express the molecular wave function of each DNA component with the GAUSSIAN09 software^31^. Gaussian 09 is a general purpose computational chemistry software, which optimizes by variational principle, i.e. Hartree-Fock (HF) method, the ground state of atoms and molecules. Thus, DNA components ground states have been obtained by a Restricted Hartree-Fock (RHF) optimization using the 3–21G basis set. Each target is described via N molecular subshell wave functions with N = 35, 29, 39, 33 and 48 molecular orbitals (MOs) for adenine, cytosine, guanine, thymine and sugar-phosphate backbone unit, respectively. Thus, the molecular subshell wave function is expressed as a linear combination of atomic wave functions corresponding to the different atomic components, namely, H_1*s*_, C_1*s*_, C_2*s*_, C_2*p*_, N_1*s*_, N_2*s*_, N_2*p*_, O_1*s*_, O_2*s*_, O_2*s*_, P_1*s*_, P_2*s*_, P_3*s*_, P_3*p*_, and P_3*p*_. For each MO, the effective number of electrons relative to the atomic component was derived from a standard Mulliken population analysis and their sum for each occupied MO is very close to 2^32^. Besides, the computed ionization energies of the occupied MOs of the biological targets investigated here were scaled so that their calculated Koopmans ionization energy, i.e. the ionization energy of their HOMO, coincides with experimental values available in the literature (for more details, we refer the interested reader to our previous study^30^).

In order to get a more realistic description of the biological matter at nano-scale level, a typical nucleotide has been considered here, i.e. an equivalent unit of DNA molecule composed of a nucleobase-pair plus two sugar-phosphate groups^33^. To fit the realistic composition of living cells, the nucleobase repartition percentages reported by Tan *et al*.^34^, namely, 58% (A-T) (adenine-thymine base pair) and 42% (C-G) (cytosine-guanine base pair) were considered. Thus, by using the respective molar mass of each DNA component, namely, M_A_ = 135.14 g mol^−1^, M_T_ = 126.12 g mol^−1^, M_C_ = 111.11 g mol^−1^, M_G_ = 151.14 g mol^−1^ and M_sug−phos_ = 180 g mol^−1^, the following mass percentages were obtained: A (12.6%), T (11.8%), C (7.5%), G (10.2%) and sugar-phosphate group (57.9%). This description refers to dry DNA, which obviously cannot mimic the biological reality, mainly composed of hydrated DNA. Consequently, we investigated a biological medium composed of hydrated DNA, where 18 molecules per nucleotide have been added, which led to the following revisited mass percentages: A (8.3%), T (7.7%), C (4.9%), G (6.7%), sugar-phosphate group (38.1%) and water (34.3%). This result was obtained following the work of Birnie *et al*.^35^, who estimated that the total amount of water associated with DNA was of the order of 50 moles per mole of nucleotide, in order to get the expected density of 1.29 g cm^−3^. Finally, a typical nucleotide was modeled as 0.58 of adenine–thymine, 0.42 of cytosine–guanine, 2 groups of sugar–phosphate and 18 molecules of water, with a respective molar mass of 947.8 g mol^−1^.

### Distorted wave theories: an overview

The interaction of charged particles with matter has been a matter of study since 1930, and many theories have emerged since. In this problem all the particles involved are charged and, therefore, the interactions are governed by long-range Coulomb potentials acting even at infinitely large distances. Between the different theoretical frameworks available, the distorted wave theory is one of the best suited to board this kind of problems. Due to the fact that the Coulomb interaction between charged particles might be small at large distances but never zero, the wave function describing these systems cannot be written as the product of free-particle (plane-waves) wave functions. Also, the long-range nature of the potential gives place to the appearance of a Coulomb phase or distortion. In distorted wave theory, the initial and final state wave functions are chosen as the product of the initial and final state of the target with the corresponding initial and final channel distortions, respectively, due to the interaction with the projectile. These functions are chosen in order to represent in the best possible way the physics of the problem and to keep the correct asymptotic conditions at large distances. These wave functions can be chosen in different ways giving place to different distorted wave models.

Another basic characteristic useful to simplify the collision problem between heavy particles is the fact that the nuclei are much more massive than the electrons. In particular this mass relation between the electrons and nuclei masses allows to formulate what is known as the *impact parameter approximation* in which classical trajectories are assumed for the heavy particles. This classical evolution of the heavy particles generates a time-dependent potential in which the electrons move, the latter being treated quantically. The validity of this impact parameter approximation is assured within the range of intermediate and high collision energies (see the work by McCarroll and Salin^36^ and references therein). In particular, in the distorted wave theories developed in this work, we make use of the *straight-line version* of the impact parameter approximation, which consists in assuming that at sufficiently high collision energy the projectile describes a rectilinear trajectory defined by

10 $${\bf{R}}={\boldsymbol{\rho }}+{\bf{v}}\,t,$$

where **R** is the internuclear vector, ***ρ*** the impact parameter, **v** the impact velocity (***ρ*** ⋅ **v** = 0), and *t* the collision time.

One of the most successful theoretical descriptions is the Continuum Distorted Wave-Eikonal Initial State (CDW-EIS) model introduced by Crothers and McCann^37^ to study electron ionization of monoelectronic targets and later extended by Fainstein *et al*.^38^ for multielectronic targets. The CDW-EIS model was also formulated to investigate the electron capture reaction by Martínez *et al*.^39^.

### The continuum distorted wave–eikonal initial state model

The theoretical model employed in the present work to calculate electron emission and electron capture by proton impact is the well known Continuum Distorted Wave–Eikonal Initial State (CDW-EIS). Regarding the multielectronic nature of the molecular targets here considered, provided that the collision velocity *v* is high enough, the problem can be reduced to the analysis of a one-active electron system by considering that all the other target electrons (the passive ones) remain frozen in their initial orbitals during the collision and that the active electron evolves independently of them in an effective mean field of the residual target. The extension of the CDW-EIS theory for electron ionization of multielectronic targets was firstly introduced by Fainstein *et al*.^38,40^. In a similar way, we can consider the times associated to the vibration and rotation of the molecules to be much larger than the characteristic times of the collision. It is then possible to assume that the molecular nuclei remain fixed in their initial positions during the reaction. The CDW-EIS model is a first-order perturbative theory in which the *prior* version of the transition amplitude, within the straight-line version of the impact parameter approximation, can be written as^41^

11 $$a{({\boldsymbol{\rho }})}_{i,f}^{-}={\int }_{-\infty }^{+\infty }\,dt\langle {\chi }_{f}^{-}|{W}_{i}|{\chi }_{i}^{+}\rangle $$

where ${\chi }_{i}^{+}$ (${\chi }_{f}^{-}$) is the initial (final) channel distorted wave function satisfying outgoing (incoming) boundary conditions and *W*_*i*_ is the perturbative operator acting on the entry channel^37^. It is usual to relate $a{({\boldsymbol{\rho }})}_{i,f}^{-}$ with the transition amplitude as a function of the transverse momentum transfer^36,42^, through the relation

12 $$ {\mathcal R} {({\boldsymbol{\eta }})}_{i,f}^{-}=\frac{1}{2\pi }\int d{\boldsymbol{\rho }}\,\exp ({\rm{i}}{\boldsymbol{\eta }}\cdot {\boldsymbol{\rho }})a{({\boldsymbol{\rho }})}_{i,f}^{-}.$$

The advantage of working with the ${\mathcal R}$ transition amplitude is that it is possible to find analytical expressions for it, drastically reducing the calculation times. In this work we use the CDW-EIS model to describe the electron emission and electron capture processes.

#### The electron emission process

The CDW-EIS model has been widely used to calculate electron ionization by bare projectile impact from a grand variety of targets from mono and multielectronic atoms to large complex biomolecules^30^. The distorted wave functions ${\chi }_{i}^{+}$ and ${\chi }_{f}^{-}$ for ionization are chosen as

13 $${\chi }_{i}^{+,EIS}={\varphi }_{i}({\bf{x}})\exp (-{\rm{i}}{\varepsilon }_{i}t){ {\mathcal L} }_{i}^{+,EIS}({\bf{s}})$$

14 $${\chi }_{f}^{-,CDW}={\varphi }_{f}({\bf{x}})\exp (-{\rm{i}}{\varepsilon }_{f}t){ {\mathcal L} }_{f}^{-,CDW}({\bf{s}})$$

with *ϕ*_*i*_ (*ϕ*_*f*_) the initial bound (final continuum) active electron wave function, *ε*_*i*_ the initial bound state binding energy, and *ε*_*f*_ = *k*^2^/2 the final continuum energy, being **k** and **x** the electron momentum and position, respectively, on a target-fixed reference frame, and **s** its position on a projectile-fixed reference frame.

The eikonal and continuum distortion factors in Eqs 13 and 14 are given by

15 $${ {\mathcal L} }_{i}^{+,EIS}({\bf{s}})=\exp [-{\rm{i}}\nu \,\mathrm{ln}(v\,s+{\bf{v}}\cdot {\bf{s}})]$$

16 $${ {\mathcal L} }_{f}^{-,CDW}({\bf{s}})={N}^{\ast }(\zeta ){}_{1}{F}_{1}[-{\rm{i}}\zeta ;1;-{\rm{i}}(p\,s+{\bf{p}}\cdot {\bf{s}})]\,\,,$$

respectively, where *ν* = *Z*_*P*_/*v* and *ζ* = *Z*_*P*_/*p*, with *Z*_*P*_ the charge of the projectile and **p** = **k**−**v** the electron momentum from a projectile-fixed reference frame. In Eq. (16) _1_*F*_1_[*b*, *c*, *z*] is the Kummer confluent hypergeometric function and *N*(*a*) = Γ(1−i*a*)exp(*πa*/2) its normalization factor.

The initial-bound wave function, *ϕ*_*i*_, of each molecular orbital is considered as a linear combination of the atomic orbitals of its compounds, LCAO approximation (see the work by Tachino *et al*.^27^ and the description of the targets provided above for more details).

The emitted electron final continuum state is approximated by an hydrogenic continuum function with an effective charge, ${\tilde{Z}}_{T}$, chosen in correspondence with the initial molecular binding energy *ε*_*i*_ by ${\tilde{Z}}_{T}=\sqrt{-2{n}^{2}{\varepsilon }_{i}}$, i.e.

17 $${\varphi }_{f}({\bf{x}})=\frac{\exp ({\rm{i}}{\bf{k}}\cdot {\bf{x}})}{{(2\pi )}^{3/2}}{N}^{\ast }(\lambda ){}_{1}{F}_{1}[-{\rm{i}}\lambda ;1;-{\rm{i}}(k\,x+{\bf{k}}\cdot {\bf{x}})]\,\,,$$

with $\lambda ={\tilde{Z}}_{T}/k$. The *n* present in ${\tilde{Z}}_{T}$ is the principal quantum number of each of the atomic orbitals used to describe the molecular ones. In the electron emission reaction, the perturbative operator of Eq. (11) results

18 $${W}_{i}{\chi }_{i}^{+}=\exp (-{\rm{i}}{\varepsilon }_{i}t)[\frac{1}{2}{{\rm{\nabla }}}_{s}^{2}\,{{\mathscr{L}}}_{i}^{+}({\bf{s}})+{{\rm{\nabla }}}_{x}\,{\varphi }_{i}({\bf{x}})\cdot {{\rm{\nabla }}}_{s}\,{{\mathscr{L}}}_{i}^{+}({\bf{s}})].$$

In this way, the transition amplitude results

19 $$a{({\boldsymbol{\rho }})}_{i,f}^{-}={\int }_{-{\rm{\infty }}}^{+{\rm{\infty }}}\,dt\int d{\bf{x}}\{{\varphi }_{f}^{\ast }({\bf{x}}){{\mathscr{L}}}_{f}^{-\ast }({\bf{s}})[{\varphi }_{i}({\bf{x}})\frac{1}{2}{{\rm{\nabla }}}_{s}^{2}\,{{\mathscr{L}}}_{i}^{+}({\bf{s}})+{{\rm{\nabla }}}_{x}{\varphi }_{i}({\bf{x}})\cdot {{\rm{\nabla }}}_{s}{{\mathscr{L}}}_{i}^{+}({\bf{s}})]\}$$

The corresponding double differential cross section (DDCS) as a function of the emitted electron energy *E*_*k*_ and solid angle Ω_*k*_, can be written as

20 $$\frac{{d}^{2}\sigma }{d{E}_{k}\,d{\Omega }_{k}}=k\int d{\boldsymbol{\rho }}\,{|a{({\boldsymbol{\rho }})}_{i,f}^{-}|}^{2}=k\int d{\boldsymbol{\eta }}\,{| {\mathcal R} {({\boldsymbol{\eta }})}_{i,f}^{-}|}^{2}$$

**Figure 2:**
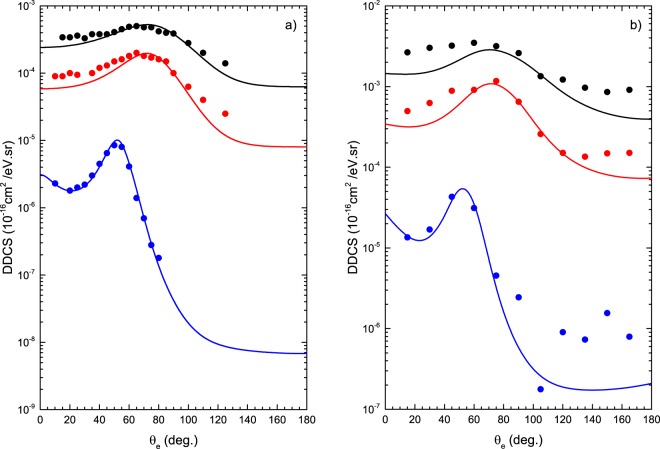
CDW-EIS DDCS for ionization induced by H^+^ in water (panel a) and in adenine (panel b). Theoretical calculations performed for 1 MeV impact energy and different ejected electron energies (solid lines): black *E*_*e*_ = 50 eV, red *E*_*e*_ = 100 eV and blue *E*_*e*_ = 750 eV (for water) while *E*_*e*_ = 700 eV (for adenine). Experimental data (circles) are taken from Toburen and Wilson^72^ (panel a) and Iriki *et al*.^73^ (panel b).

Then, by numerical integration of the DDCS, Eq. (20), over the solid angle Ω_*k*_ it is possible to obtain the singly differential cross sections (SDCS). Further, the TCS is computed by integrating Eq. (20) over the solid angle Ω_*k*_ and in electron energy *E*_*k*_. In Fig. 2, DDCS computed within the CDW-EIS approximation are reported for proton impact on water vapor and adenine molecules. The theoretical results describe well the experimental data for several emitted electron energies, *E*_*k*_, in function of the ejected emission angle *θ*_*e*_.

Finally, let us add that the case of ionization by neutral hydrogen is currently in progress and will be covered in detail in a separate paper^43^.

#### The electron capture process

The CDW-EIS has been widely used to investigate the electron capture process of various atoms impacted by bare projectiles^39,44^. Moreover, the CDW-EIS approximation has also been used to study the electron capture in ion-molecule collisions, namely, proton impact on DNA nucleobases^45^, proton^46^ and ions^47^ impact on molecules of biological interest. In the CDW-EIS approximation, the main difference between the electron emission and electron capture process is that in the latter the electron final state is not in the target continuum state, but in the projectile bound state. In this sense, the initial channel distorted wave function remains unaltered with respect to the ionization case, but the final channel one results

21 $${\chi }_{f}^{-,CDW}={\varphi }_{f,{\rm{nlm}}}({\bf{s}})\exp [-{\rm{i}}{\varepsilon }_{f}t+{\rm{i}}{\bf{v}}\cdot {\bf{x}}-{\rm{i}}\frac{1}{2}{v}^{2}t]{ {\mathcal L} }_{f}^{-,CDW}({\bf{x}}),$$

when in this case *ϕ*_*f*,nlm_ represents a final projectile bound state, namely a hydrogenic bound state with quantum numbers *n*, *l* and *m*, and binding energy ${\varepsilon }_{f}=-\,{Z}_{P}^{2}/2{n}^{2}$. The distortion factor in Eq. (21) is chosen as a target continuum factor

22 $${ {\mathcal L} }_{f}^{-,CDW}({\bf{x}})={N}^{\ast }{(\xi )}_{1}{F}_{1}[-{\rm{i}}\xi ;1;-i(v\,x+{\bf{v}}\cdot {\bf{x}})],$$

with $\xi ={\tilde{Z}}_{T}/v$, as it was previously defined in the case of ionization process. Following the result given in Eq. (12), the total cross section for electron capture is obtained as

23 $${\sigma }_{if}=\int d{\boldsymbol{\eta }}| {\mathcal R} {({\boldsymbol{\eta }})}_{if}^{-}{|}^{2}\,$$

It is worth noting that in *TILDA-V* the neutral hydrogen induced electron capture process, which leads to the formation of the negative H^−^ ion is neglected. The rationale behind this decision is that the cross sections for the production of H^−^ are at least one order of magnitude smaller than those corresponding to the hydrogen-induced electron loss process, as reported by Abicht *et al*.^48^.

### The electron loss process

The electron loss process is taken into account in *TILDA-V* following the approach developed by Miller and Green^49^. Starting from experimental cross sections^50,51^, the authors fitted the measurements using the expression

24 $${\sigma }_{{\rm{eloss}}}({E}_{{\rm{inc}}})={a}_{0}\frac{{(Za)}^{\Omega }{({E}_{{\rm{inc}}}-W)}^{\nu }}{{J}^{\Omega +\nu }+{E}_{{\rm{inc}}}^{\Omega +\nu }}$$

with *a*_0_ = 10^−16^ cm^2^, *Z* the number of electrons in the target molecule (for the water molecule *Z* = 10), *E*_inc_ being the impact energy and *W* the ionization threshold. *ν* and Ω are dimensionless parameters, whereas *a* and *J* are parameters with the dimension of energy. Using Eq. (24) the authors obtained the best fit with the following values^49^ *a* = 79.3 keV, *J* = 27.7 keV, Ω = 0.652, and *ν* = 0.943. In the case of the DNA components, neither experimental data nor theoretical predictions are available. Thus, the total electron loss cross sections are obtained by applying a simple rescaling procedure. Starting from the TCS of water vapor, the TCS for a given DNA component is computed as

25 $${\sigma }_{{\rm{e}}{\rm{l}}{\rm{o}}{\rm{s}}{\rm{s}}}^{({\rm{D}}{\rm{N}}{\rm{A}}{\rm{c}}{\rm{o}}{\rm{m}}{\rm{p}}{\rm{o}}{\rm{n}}{\rm{e}}{\rm{n}}{\rm{t}})}({E}_{{\rm{i}}{\rm{n}}{\rm{c}}})=TC{S}^{({\rm{w}}{\rm{a}}{\rm{t}}{\rm{e}}{\rm{r}})}({E}_{{\rm{i}}{\rm{n}}{\rm{c}}})\,\frac{{Z}^{{\rm{^{\prime} }}}}{Z}$$

with *Z* = 10 the number of electrons of the water molecule and *Z*′ the number of electrons of a given DNA component (adenine *Z*′ = 70, cytosine *Z*′ = 58, thymine *Z*′ = 66, guanine *Z*′ = 78 and sugar-phosphate *Z*′ = 96). The rescaling procedure adopted in the present work, which is based on the number of target electrons, was suggested by the work of Champion *et al*.^52^, where they have shown that the total cross sections, for the ionization and electron capture processes, are directly linked to the total number of target electrons.

### The electronic excitation process

The MC codes developed in the last decades are mainly based on the semi-empirical approach proposed by Miller and Green^49^ for describing the excitation process. This approximation assumes a velocity scaling of the electron-induced excitation cross sections together with extensions towards lower proton energies (see for example the work by Uehara *et al*.^53^). In their model, the authors reported a large set of fitting parameters for simulating the proton-induced excitation cross sections for 28 excited states of the water molecule.

In *TILDA-V*, an extension of this model described by Dingfelder *et al*.^21^ was adopted, who suggested a slightly modified set of parameters so that the semi-empirical approach agrees with the 1st Born approximation predictions towards the high-energy limit. In Table 3 we have reported the fitting parameters proposed by Dingfelder *et al*.^21^, which allow us to compute the excitation cross section of the water molecule (*Z* = 10) impacted by protons using Eq. (24).

**Table 3:** Parameters for the excitation cross section.

Excited State	*W*(eV)	*a*(eV)	*J*(eV)	Ω	*ν*
${\tilde{A}}^{1}{B}_{1}$	8.17	876	19820	0.85	1
${\tilde{B}}^{1}{A}_{1}$	10.13	2084	23490	0.88	1
Ryd A + B	11.31	1373	27770	0.88	1
Ryd C + D	12.91	692	30830	0.78	1
Diffuse bands	14.50	900	33080	0.78	1

For the neutral hydrogen impact on the water molecule, the excitation cross section could be determinated by the same analytical expression, Eq. (24), as in the case of proton impact. Thus, we have followed the approach of Uehara *et al*.^53^, who assumed that in water one of the fitting parameters is changed, namely, the parameter *a* is equal to 3/4 of the proton value (see Table 3).

For DNA components, a similar procedure of velocity scaling of electron-induced excitation cross sections, as that employed by Miller and Green^49^, was followed. Starting from poor available experimental TCS of electron-induced excitation of DNA components (essentially on thymine), we have adopted the analytical method proposed by Miller and Green to extrapolate the fitting parameters needed in the semi-empirical formula, Eq. (24), for modeling the proton-induced excitation^15^. To compute the neutral hydrogen excitation cross sections for DNA components we have adopted the same consideration as in the water case^15^.

### The elastic scattering process

The proton-induced elastic scattering cross sections are computed from the classical mechanical theory. In this context, the classical differential cross section for the elastic scattering of protons is given by the well-known relation

26 $$\frac{d\sigma }{d\Omega }=-\,\frac{\rho }{\sin \,\theta }\frac{d\rho }{d\theta },$$

where *ρ* refers to the impact parameter and *θ* denotes the scattering angle in the center-of-mass (CM) system defined by

27 $$\theta =\pi -2{\int }_{{r}_{{\rm{\min }}}}^{\infty }\,\frac{\rho }{{r}^{2}\sqrt{1-V(r)/{E}_{{\rm{inc}}}^{{\rm{CM}}}-{\rho }^{2}/{r}^{2}}}dr,$$

where *r*_min_ is the distance of closest approach and ${E}_{{\rm{inc}}}^{{\rm{CM}}}$ is the incident particle energy in the center-of-mass system. In Eq. (27), let us note that the interaction potential *V*(*r*) is analytically calculated from the wave function of the medium of interest (water or DNA component).

The elastic TCS are then obtained by numerical integration of the SDCS over the scattering solid angle by using a cutoff angle in order to reduce the divergence due to the high values of the SDCS at low scattering angles.

Finally, let us note that for the elastic scattering of neutral hydrogen atoms on water molecules we followed the suggestion of Endo *et al*.^54^ and fitted the hydrogen vs. proton cross section ratio to

28 $$\frac{{\sigma }_{{H}^{0}}}{{\sigma }_{{H}^{+}}}=1+0.0224\,\log ({E}_{{\rm{inc}}})+0.01285\,\log \,{({E}_{{\rm{inc}}})}^{2},$$

which leads to a ratio close to unity within the incident energy range considered here.

Work is in progress to compute the elastic scattering cross sections for DNA.

### Total cross sections of the physical processes in *TILDA-V*

**Figure 3:**
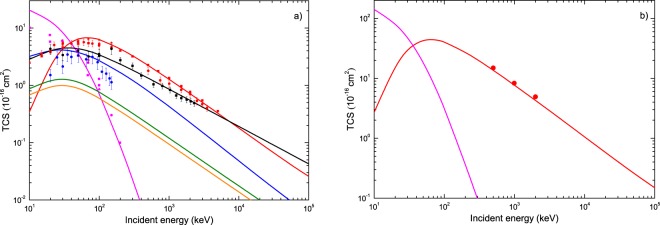
TCS for H^+^ and H^0^ in water (panel a) and for H^+^ in adenine (panel b). Theoretical CDW-EIS and semi-empirical predictions (solid lines): ionization (red), capture (magenta) and excitation (green) induced by proton impact; ionization (blue), electron loss (black) and excitation (orange) induced by hydrogen. Experimental data for water are taken from Rudd *et al*.^74^, Bolorizadeh *et al*.^75^ and Luna *et al*.^76^ for proton-induced ionization; from Toburen *et al*.^50^, Dagnac *et al*.^51^ and Gobet *et al*.^77^ for electron capture induced by proton; from Gobet *et al*.^77^ and Barnett *et al*.^78^ for ionization and electron loss induced by neutral hydrogen. Experimental data for adenine are taken from Iriki *et al*.^73^.

The TCS for all the above-described physical processes are depicted in Fig. 3. For water vapor impacted by protons (Fig. 3a), we report the obtained TCS for all the six physical processes, namely, ionization, electron capture and excitation induced by proton, and ionization, electron loss and excitation induced by neutral hydrogen. It can be observed that in the case of ionization and electron capture induced by protons (red and magenta, respectively), the theoretical predictions of the TCS, computed within the CDW-EIS approximation, are well in agreement with the various sets of measurements for impact energies greater than 40 keV. For energies below 40 keV, the CDW-EIS results underestimate the ionization cross section, whereas for the electron capture process the theoretical results are greater than the experimental data at least by a factor of 2. For the ionization induced by neutral hydrogen (blue), the theoretical TCS computed within the CDW-EIS approximation are in accordance with reported experimental data. In Fig. 3b we report the CDW-EIS theoretical prediction of TCS for ionization (red) as well as for electron capture (magenta) induced by protons on the adenine molecule. For ionization, the obtained results show a good agreement with the experimental data. Whereas for electron capture, only theoretical results are reported since no experimental data currently exist.

**Figure 4:**
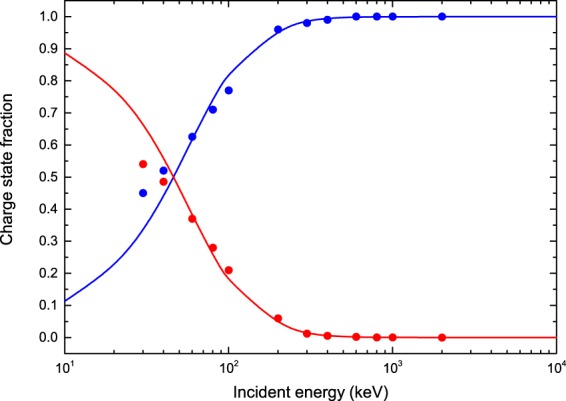
Equilibrium charge-state fractions of proton and hydrogen beams in water vapor as a function of the impact energy. Theoretical prediction CDW-EIS: H^+^ charge-state fraction (solid blue line); H^0^ charge-state fraction (solid red line). Experimental data are taken from Barnett *et al*.^78^ (solid circles).

As it is shown in Fig. 3a, in the low-energy region, below 50 keV, the electron capture process becomes more important than the ionization one. As a consequence, during the continuous slowing-down of the proton beam, a fraction of protons in the beam will change of state when its energy approaches the Bragg’s peak region. In Fig. 4, we report the charge-state fractions, as given in Eqs 3 and 4, considering the TCS of electron capture (CDW-EIS) and the TCS of electron loss (semi-empirical). The theoretical equilibrium charge-state fractions show a good agreement with the experimental measurements for impact energies greater than 60 keV.

## Results and Discussion

Proton tracking simulations were performed with *TILDA-V*. In this section we present the results obtained for the inelastic mean free path, the electronic stopping power and the range for protons in water and DNA and compare our predictions with available data. Then we report the results regarding the irradiation of individual cells by protons.

### Proton track structure in water and DNA

**Figure 5:**
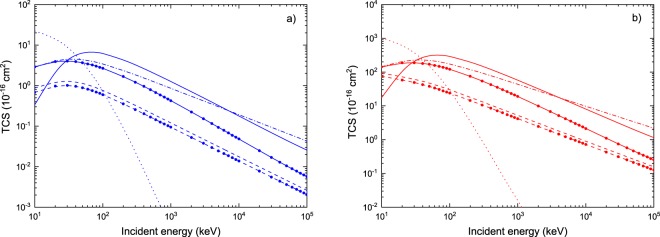
Inelastic TCS for H^+^ and H^0^ in water (panel a) and hydrated DNA (panel b). In both cases, the TCS for ionization by H^+^ (solid line), ionization by H^0^ (solid line with circles), excitation by H^+^ (dashed line), excitation by H^0^ (dashed line with circles), electron capture (dotted line) and electron loss (dash-dotted line) are shown.

Figure 5 shows the inelastic TCS for protons (H^+^) and neutral hydrogen atoms (H^0^) impinging on the water (panel a) and DNA (panel b) targets. A difference of almost two orders of magnitude is observable between the TCS of both media. However, let us remind that hydrated DNA is considered here, which already contains the contribution of 18 water molecules. Therefore, it is not surprising to find TCS values for DNA much greater than those for water. A more meaningful comparison of the targets can be made through the IMFP. We have applied Eq. (5) to compute the IMFP for protons in different media over the incident energy range of 10 keV to 100 MeV.

**Figure 6:**
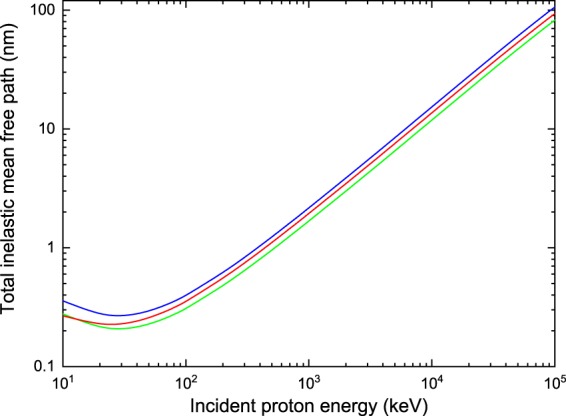
Total IMFP for protons in water (blue line), water with a density rescaled to 1.29 g cm^−3^ (green line) and hydrated DNA (red line) as a function of the incident energy.

Figure 6 shows the total IMFP for protons in water (*ρ* = 1.0 g cm^−3^, blue line), water with a density rescaled to 1.29 g cm^−3^ (green line) and hydrated DNA (*ρ* = 1.29 g cm^−3^, red line). We would like to emphasize that in the results presented in Fig. 6, all the inelastic processes and charge-states previously discussed in the Theory section have been taken into account, namely ionization, excitation and electron capture by proton, and ionization, excitation and electron loss by neutral hydrogen. It is noticeable from Fig. 6 that the IMFP for protons in water and DNA takes distinct values at all incident energies, the values for water being, on average, about 13% greater that the ones for DNA. More important, however, is the fact that applying a simple density rescaling to the TCS of water is not enough to reproduce the behavior of protons in DNA, as it can be seen from the green curve in Fig. 6.

**Figure 7:**
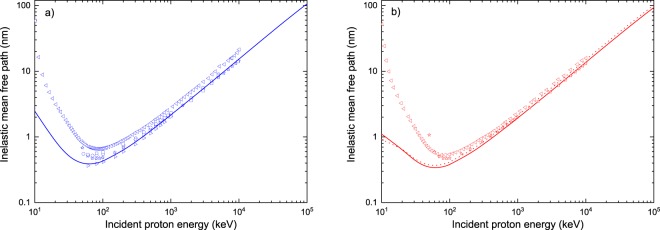
IMFP for protons in water (panel a) and DNA (panel b), as obtained in this work (solid lines), when only ionization and excitation by protons are taken into account. The symbols correspond to calculations performed by Dingfelder *et al*.^21^ (squares), Uehara *et al*.^53^ (right-pointing triangles), Emfietzoglou *et al*.^55^ (circles), Tan *et al*.^56^ (stars) and de Vera *et al*.^57^ (left-pointing triangles). The dotted line in panel b) shows the predictions of *TILDA-V* when considering the same DNA composition as Tan *et al*.^56^, as explained in the text.

Besides, to compare our predictions with other works, we have plotted in Fig. 7 the IMFP for protons considering only the role of the ionization and excitation processes induced by H^+^. The results for water are depicted in Fig. 7a and compared with available data taken from Dingfelder *et al*.^21^ (squares), Uehara *et al*.^53^ (right-pointing triangles), Emfietzoglou *et al*.^55^ (circles), Tan *et al*.^56^ (stars) and de Vera *et al*.^57^ (left-pointing triangles). For incident proton energies *E*_inc_ > 1 MeV, all calculations converge, except for those of de Vera and co-workers^57^ which remain about 25% greater. At lower incident energies, i.e. from 300 keV to 1 MeV, the difference between our results and those of refs^21,53,55,56^ is within 10%. What is more, the best agreement at low energies is observed with the calculations of Uehara and co-workers^53^. This is understandable because they considered water vapor targets as well, and it is well-known that phase effects become important as the projectile energy decreases. All other data sets reported in Fig. 7a correspond to liquid water. In addition, significant deviations arise between the various calculations for ${E}_{{\rm{inc}}}\lesssim 300$ keV. This is not surprising, since all the models have limitations in the low-energy region. The condensed phase approaches implemented in refs^55–57^ are valid above some hundreds of keV, while the CDW-EIS predictions are considered reliable above ~40 keV (see Fig. 3). Figure 7b depicts the IMFP for protons in DNA. The solid line represents our results for hydrated DNA. Data from Tan *et al*.^56^ (stars) and de Vera *et al*.^57^, who worked with dry DNA, are shown for comparison. Overall, a very similar behavior to the one reported in Fig. 7a is observed for DNA. An excellent agreement is found between our predictions and the calculations of Tan and co-workers^56^ for high incident energies, i.e. for *E*_inc_ > 1 MeV, with differences below 4%; the deviations with respect to the values obtained by de Vera and co-workers^57^ in that same energy range are greater (~19%), but they are smaller than the differences reported above for water (~25%). These discrepancies grow for decreasing values of the incident energy, reaching about 25% and 40% at 100 keV with respect to the work of Tan *et al*.^56^ and de Vera *et al*.^57^, respectively. Once more, let us note that the predictions for energies below some hundreds of keV, and for ${E}_{{\rm{inc}}}\lesssim 40$ keV in the case of the CDW-EIS model, are questionable. Furthermore, to see if a better agreement could be reached by considering dry DNA instead of hydrated DNA, we adopted the same DNA composition as Tan *et al*.^34,56^. To do so, we subtracted the contribution of the water molecules to the DNA TCS and used in our calculations a density of 1.35 g cm^−3^ and a molar mass of 662 g mol^−1^, as reported by Tan *et al*.^34^. The results are represented by the dotted line in Fig. 7b. A better agreement is indeed achieved, but only for 200 keV < *E*_inc_ < 1 MeV. Thus, the remaining discrepancies between the dotted line and the data of Tan *et al*.^56^ should be ascribed to the inherent differences in the models employed to compute the TCS, and not to the DNA description.

**Figure 8:**
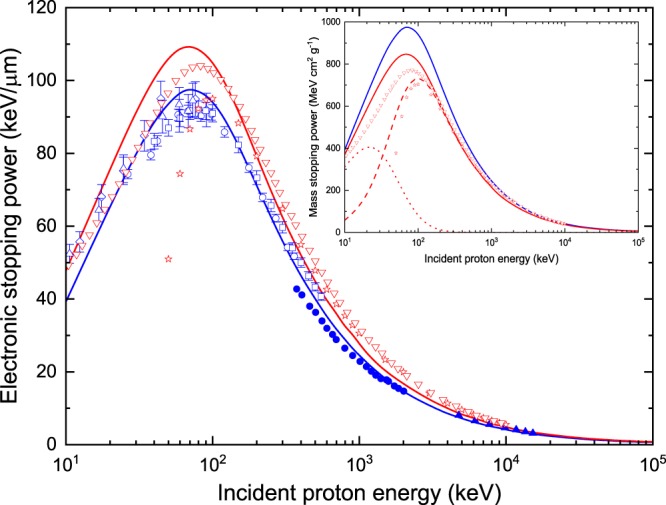
Total electronic stopping power for protons in water (solid blue line) and DNA (solid red line). Experimental data for water vapor are shown in open blue symbols and are taken from: Reynolds *et al*.^79^ (squares), Phillips^80^ (triangles), Mitterschiffthaler and Bauer^81^ (circles) and Baek *et al*.^82^ (diamonds). Measurements for liquid water are shown in solid blue symbols and are taken from: Shimizu *et al*.^58^ (circles) and Siiskonen *et al*.^59^ (triangles). For DNA the calculations performed by Abril *et al*.^22^ (open inverted red triangles) and Tan *et al*.^34^ (open red stars) are shown for comparison. The inset depicts the mass electronic stopping power for protons in water (blue line) and DNA (solid red line). The dashed and dotted red lines indicate the H^+^ and H^0^ contributions to the stopping power for protons in DNA. The symbols correspond once again to the calculations of Abril *et al*.^22^ (open inverted red triangles) and Tan *et al*.^34^ (open red stars).

Figure 8 presents the total electronic stopping power for protons in water and DNA, as provided by *TILDA-V*. Our results for water have been compared with the available experimental data for both water vapor and liquid water. In general, a good agreement can be observed between our predictions for water and the experimental data, especially for energies above 30 keV. More specifically, for *E*_inc_ > 30 keV our predictions fall most of the time within the estimated errors in the reported measurements for water vapor, with deviations of less than 10%. For lower energies, however, our values underestimate the experimental results, a behavior that can be attributed to the theoretical model implemented in our code (*prior* CDW-EIS, see Table 1). Furthermore, when comparing our predictions with the available experimental data on liquid water, we found that our results are greater than the ones reported by Shimizu *et al*.^58^ (full circles) by about 5–13%. On the other hand, our values are actually smaller than those obtained by Siiskonen *et al*. (full triangles)^59^, but it should be noted that the relative differences are only slightly above the total uncertainty reported by the authors (4.6%). It is important to recall that *TILDA-V* simulations are based on cross sections computed in water vapor; correspondingly, the stopping power results presented here were obtained by using the ionization potentials for water vapor. In condensed media, such as liquid water, it is known that the stopping power values are smaller than in the gas phase as a result of the polarization of the medium by the charged projectile. We have observed that if the ionization potentials for liquid water (as provided by Dingfelder *et al*.^21^) were used instead to calculate the stopping power, while keeping the same cross sections, the results would be smaller than the ones shown in Fig. 8 (solid blue line) by about 8% at 10 keV, 4% at 100 keV, and 2–3% for energies up to 100 MeV. The latter, although a very simple test, shows that for liquid water smaller stopping power values would be obtained, as expected from the polarization effect. For comparison, the difference between the mass stopping power values in vapor and liquid water according to the PSTAR database^60^ are of 13.8% at 10 keV, 12.6% at 100 keV, 1.4% at 1 MeV, 0.8% at 10 MeV and 0.6% at 100 MeV, rising again in the GeV region.

In the case of DNA, because of the lack of experimental data, we have included in Fig. 8 the few theoretical data found in the literature, namely the calculations made by Abril *et al*.^22^ and Tan *et al*.^34^. Both research groups considered dry DNA with a density of 1.35 g cm^−3^ and performed their calculations within the dielectric formalism, but using different extension schemes. Clearly, there are important differences between our results and their predictions for incident energies below 200 keV.

The inset in Fig. 8 shows the mass electronic stopping power for protons in water (blue line) and DNA (solid red line), obtained by dividing the stopping power by the density of the medium. Regarding DNA, we have plotted the H^+^ and H^0^ contributions to the stopping power (dashed and dotted red lines, respectively). It can be observed that the neutral hydrogen contribution dominates at low incident energies (*E*_inc_ < 30 keV); on the contrary, for *E*_inc_ > 300 keV, only the contribution of H^+^ remains relevant. We have included on that graph as well the calculations by Abril *et al*.^22^ and Tan *et al*.^34^. From this graph it is clear that a better agreement is reached between our DNA results and theirs when only the H^+^ contribution to the stopping power is taken into account (i.e. frozen-charge approximation). The agreement with both authors is within ~3% at 100 keV, while once more the deviations become noticeable at lower incident energies as the models go beyond their limit of validity. Nevertheless, it should be stressed that ignoring the charge-exchange processes, i.e. the H^0^ contribution, is expected to underestimate the stopping power in the low-energy regime.

Additionally, let us mention that according to our calculations, the maximum stopping power for water (97.5 keV/*μ*m) and DNA (110 keV/*μ*m) is located at the same incident proton energy, namely 70 keV, with a difference of about 13% between both media.

**Figure 9:**
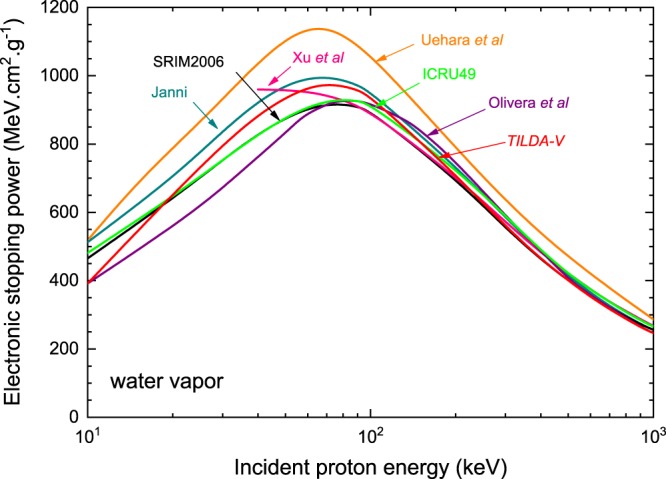
Comparison of the mass electronic stopping power for protons in water vapor, as predicted by *TILDA-V*, with other calculations and Monte Carlo codes: this work (red line), Uehara *et al*.^7^ (orange line), SRIM2006^61^ (black line), Xu *et al*.^64^ (pink line), Olivera *et al*.^65^ (purple line), ICRU Report 49^62^ (green line) and Janni^63^ (dark cyan line).

Figure 9 presents a comparison of the mass stopping powers for protons in water vapor computed with *TILDA-V* and other calculations found in the literature for incident energies ranging from 10 keV to 1 MeV.

The best agreement of our results is found with SRIM2006^61^, the ICRU Report 49^62^ and Janni’s tabulation^63^ with differences of less than 7% for 10 keV < *E*_inc_ < 1 MeV. However, the position of the maximum is slightly shifted to the right in the case of SRIM2006^61^ and the ICRU Report 49^62^ results, which reported a maximum stopping power value of ~916 MeV cm^2^ g^−1^ and ~930 MeV cm^2^ g^−1^, respectively, at 80 keV. On the other hand, the calculations of Uehara *et al*.^7^ show large discrepancies over the whole energy range. This probably stems from the fact that Uehara and co-workers^7^ computed the energy transfer during the ionization process based on average binding and emission energies. Furthermore, the calculations of Xu *et al*.^64^ performed within a modified local-plasma model are in good agreement with all the results for *E*_inc_ > 100 keV, although they predicted the stopping power maximum at lower energies. Finally, we found discrepancies of less than ~18% between our results and those of Olivera *et al*.^65^ for incident energies lower than 100 keV.

**Figure 10:**
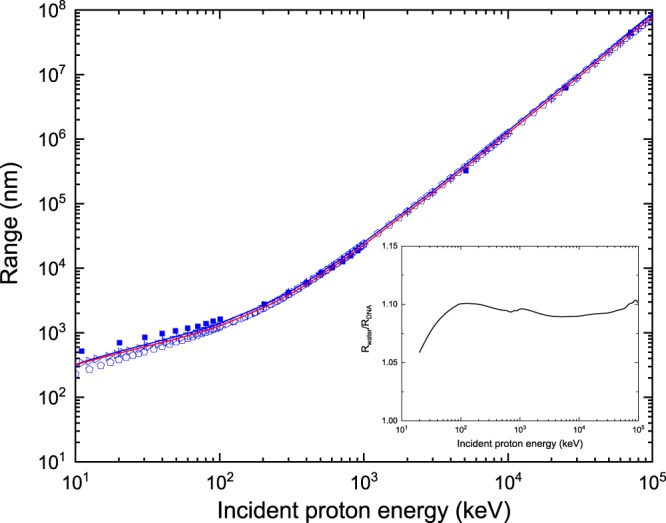
Range for protons in water (solid blue line) and DNA (solid red line) as provided by *TILDA-V*. Theoretical data for water are taken from: the ICRU Report 49^62^ (blue crosses), Uehara *et al*.^7^ (open right-pointing triangles), Janni^63^ (open pentagons) and Francis *et al*.^66^ (solid squares). The inset shows the ratio R_water_/R_DNA_, where R_water_ (R_DNA_) refers to the proton range in water (DNA).

Figure 10 represents the range for protons in water versus DNA, as provided by the latest version of *TILDA-V*. It should be noted that due to the low-energy cutoff for protons, the range values computed by *TILDA-V* must be corrected to compensate the path length that a primary particle of 10 keV would traverse until it is fully stopped in the medium. For this reason, the results presented in Fig. 10 have been obtained by adding to the output of *TILDA-V* the CSDA range for protons of 10 keV in water vapor recommended in the ICRU Report 49^62^, which is ~322 nm. This has been done for both water and DNA, since for the latter there is no equivalent data. The effect of this correction becomes negligible above 1 MeV because it represents a deviation of less than 1% in the range values. Our results have been compared with calculations and theoretical data for water taken from the ICRU Report 49^62^ (blue crosses), Uehara *et al*.^7^ (open right-pointing triangles), Janni^63^ (open pentagons) and Francis *et al*.^66^ (solid squares). The divergences between our predictions and the other models become noticeable in the low-energy regime, i.e. when *E*_inc_ < 100 keV. The greatest discrepancy is of about ~30% for *E*_inc_ = 10 keV and is found with respect to the values reported by Francis *et al*.^66^, who used the GEANT4-DNA Monte Carlo code, which as already mentioned considers liquid water. The inset in this figure shows the ratio of the proton range in water to the one in DNA as a function of the incident energy. It can be observed that the range for protons in DNA is always shorter than in water. There is a 10% difference between the range values obtained in both media, which remains more or less constant for incident energies above 100 keV. For lower incident energies, the ratio gradually decreases to about 6%.

### Microdosimetry of cell proton irradiation

**Figure 11:**
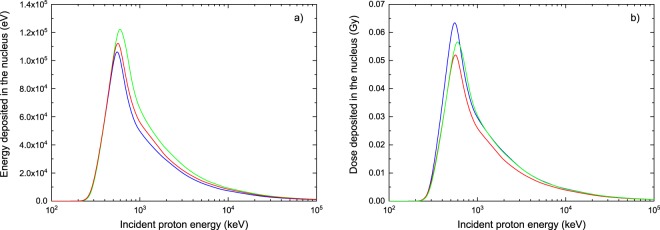
Energy (panel a) and dose (panel b) deposited in the nucleus of a single cell as a function of the incident proton energy for the configuration illustrated in Fig. 1 and when the nucleus is composed of: water (*ρ* = 1 g cm^−3^, blue line), water with a density rescaled to 1.29 g cm^−3^ (green line) and hydrated DNA (*ρ* = 1.29 g cm^−3^, red line).

The energy deposited in the nucleus of individual cells as a result of the irradiation by monoenergetic proton beams for the configuration depicted in Fig. 1 is reported in Fig. 11a. The blue, green and red lines correspond to the energy deposited in the nucleus when it is composed of water (*ρ* = 1 g cm^−3^), water with a density rescaled to 1.29 g cm^−3^ and hydrated DNA (*ρ* = 1.29 g cm^−3^), respectively. Let us remind that in our simulations the cell radius and nuclear radius were fixed to 7 *μ*m and 4 *μ*m, respectively. In other words, a proton must traverse at least 3 *μ*m of water before reaching the nucleus. The fact that the energy deposited in the nucleus is zero for incident proton energies below ~225 keV is then consistent with the proton range in water, which is of 2.99 *μ*m at that energy, according to the ICRU Report 49^62^. Additionally, the maximum energy deposit occurs for an incident energy of 550 keV in the case of water and DNA, while for density-rescaled water the peak is located at 600 keV. In the light of our results for the proton range in each medium (see Fig. 10), protons having these initial energies are able to penetrate inside the nucleus, but once there they do not have enough energy left to go out, thus they deposit all the remaining energy in that structure. It is clear from Fig. 11a that, on the one hand, modeling the cell nucleus as water may underestimate the energy deposit up to about 16% if compared to DNA, depending on the incident proton energy. This observation agrees well with the results of a previous work by Champion *et al*.^52^, who calculated the mean energy deposited by protons in cylindrical targets of DNA dimensions and found that the energy transfers were about 15% higher when DNA was used instead of water to model the medium. On the other hand, modeling the nucleus by rescaling the water density to 1.29 g cm^−3^ would have the opposite effect, namely it may overestimate the energy deposit up to about 28%, if compared to a description of the biological medium based on hydrated DNA. Finally, let us emphasize that neither the values computed with the common approach of using water as a soft-tissue surrogate nor the results obtained by applying a correction based solely on a density rescaling reflect the underlying complexity of the biological medium.

From the point of view of radiotherapy, however, it is more appropriate to express our results in terms of the total dose deposited in the nucleus (see Eq. (8)). The corresponding results are reported in Fig. 11b. It can be seen that reasoning in terms of dose provides us with a quite different perspective about the irradiated media, since now we clearly observe an overestimation of the dose deposited in the cell nucleus when water is used instead of DNA for describing the nucleus composition.

In order to better appreciate this fact, the nuclear dose ratios of water to DNA (${{\rm{D}}}_{{{\rm{H}}}_{2}{\rm{O}},1}/{{\rm{D}}}_{{\rm{DNA}},1.29}$) and of density-rescaled water to DNA (${{\rm{D}}}_{{{\rm{H}}}_{2}{\rm{O}},1.29}/{{\rm{D}}}_{{\rm{DNA}},1.29}$) are given in Table 4 for selected values of the incident proton energy. Thus, the dose deposited in water is 26% greater than in DNA at 500 keV, but this percentage drops to less than 15% for incident energies above 1 MeV. With respect to the second ratio, the deposited dose in density-rescaled water is only 1% greater than in DNA at 500 keV, this difference increases to about 28% at 700 keV and then decreases again for higher incident energies, without surpassing 18% in the energy range 1 MeV–100 MeV.

**Table 4:** Nuclear dose ratios for some values of the incident proton energy.

Incident proton energy (keV)	${{\rm{D}}}_{{{\rm{H}}}_{2}{\rm{O}},1}$/${{\rm{D}}}_{{\rm{DNA}},1.29}$	${{\rm{D}}}_{{{\rm{H}}}_{2}{\rm{O}},1.29}$/${{\rm{D}}}_{{\rm{DNA}},1.29}$
500	1.26	1.01
600	1.19	1.13
700	1.15	1.28
800	1.14	1.22
900	1.13	1.19
1000	1.15	1.18

${{\rm{D}}}_{{{\rm{H}}}_{2}{\rm{O}},1}$, ${{\rm{D}}}_{{{\rm{H}}}_{2}{\rm{O}},1.29}$ and ${{\rm{D}}}_{{\rm{DNA}},1.29}$ denote the dose deposited in the cell nucleus when it is composed of water (*ρ* = 1 g cm^−3^), water with a density rescaled to 1.29 g cm^−3^ and hydrated DNA (*ρ* = 1.29 g cm^−3^), respectively.

**Figure 12:**
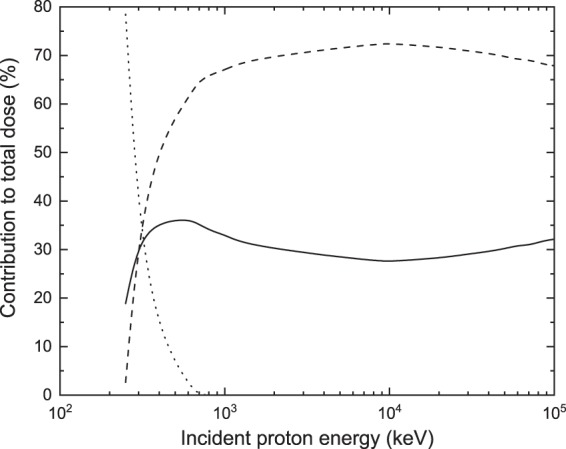
Relative contributions to the dose deposited in the cell nucleus as a function of the incident proton energy, when the nucleus is modeled by hydrated DNA. The solid, dashed and dotted lines represent the proton, neutral hydrogen and secondary electrons contribution, respectively.

Finally, we have plotted in Fig. 12 the relative contribution of the primary and the secondary particles to the nuclear dose, when the nucleus is composed of hydrated DNA. At low incident energies, the contribution to the dose is largely dominated by the neutral hydrogen (H^0^, dashed line), while the contribution of protons (H^+^) and electrons is much lower (less than 19% and 3% at an incident energy of 250 keV, respectively). This arises from the fact that the low-energy proton has lost most of its energy while traversing the cell membrane and the cytoplasm as it approaches the nucleus. Thus, its energy falls enough to enter the energy region where the electronic capture process becomes more relevant (below 100 keV), the projectile becoming H^0^ and interacting in such charge state most of the time until its remaining energy falls below the tracking cutoff of 10 keV. In the case of protons arriving in the nucleus with slightly higher energies, that is for protons with an initial energy of about 300 keV, the three contributions to the dose are almost equal. If the incident energy continues to increase, the secondary electrons provide the most important contribution to the dose. This also stems from the TCS behavior, since ionization by H^+^ becomes the most probable interaction. Let us note that for incident energies greater than 700 keV the contribution of H^0^ to the dose is essentially zero, and the secondary electron and H^+^ contributions remain more or less constant providing around 65–72% and 28–35% of the total nuclear dose, respectively. Let us add that we have only included in Fig. 12 the case of hydrated DNA because we did not find any significant variation in these contributions when the nucleus is modeled with the other media.

Lastly, let us remind that nuclear non-elastic reactions are not considered in the latest version of *TILDA-V*. These interactions take place when a proton overcomes the Coulomb barrier and enters the nucleus of one of the atoms in the target. As a result of this mechanism, primary protons are removed from the beam and the transformed nucleus may emit secondary protons, neutrons, photons and heavier ions^67,68^. Although the TCS of nuclear non-elastic reactions are several orders of magnitude lower than those of the electronic processes in the energy range considered here^67^, their effect in the computed doses may not be negligible. As a matter of fact, it is well-known that nuclear non-elastic interactions have a large influence in proton beam depth-dose distributions and are responsible for a decrease in the amplitude of the Bragg peak and an increase in the entrance dose^69–71^. Medin and Andreo^69^ observed that the removal of primary protons from the incident beam reduces the peak to entrance dose ratio by up to 40% for a 250 MeV proton beam in water. Similarly, Wroe *et al*.^71^ reported a decrease in the peak to entrance dose ratio of ~5% and ~34% for 60 and 200 MeV protons in water, respectively, when non-elastic reactions were taken into account in their simulations. It is not, however, straightforward to quantify the precise impact that the omission of the nuclear non-elastic reactions would have in our results.

## Conclusions

A reliable description and modeling of charged particle interactions in biological media is of interest for radiobiology and medicine. MCTS codes such as *TILDA-V* are the only simulation tools able to reproduce the physics of ionizing radiation at low energies and at the nanometric scale. Interdisciplinary efforts are warranted both for obtaining enough empirical data to validate the accuracy of these tools and for promoting their use in medical research.

In this work, we have reported our results for the stopping power and range of protons in water and DNA with the purpose of providing a more realistic description of the biological environment. The predictions of our code agree well with the available experimental data and theoretical calculations found in the literature in most of the proton energy range here considered (10 keV–100 MeV), but significant differences are still noticeable at low energies.

Moreover, a first study using *TILDA-V* to simulate the irradiation of individual cells by protons has been presented. Our results indicate that water and DNA do not respond to radiation in the same way and that a density rescaling procedure is not enough to take into account the biological reality. More specifically, modeling a critical cell structure such as the nucleus as composed by water only may lead to a significant overestimation of the deposited dose.

Ongoing work is devoted to the extension of *TILDA-V* to simulate the He^*q*+^ (*q* = 0, 1, 2) ion transport in a biological medium. This will allow to perform similar studies with *α* particles, in particular with the spectra of some *α*-emitting radionuclides regarded as promising for targeted alpha therapy.
